# The role of artificial intelligence in diagnosing pediatric dental disorders—a narrative review

**DOI:** 10.3389/fdmed.2025.1685359

**Published:** 2026-02-02

**Authors:** Prathibha Prasad

**Affiliations:** College of Dentistry, Ajman University, Ajman, United Arab Emirates

**Keywords:** artificial intelligence, pediatric dentistry, machine learning, deep learning, delivery of health care

## Abstract

Artificial Intelligence (AI) is revolutionizing healthcare, and its application in pediatric dentistry is showing significant promise in improving diagnostic accuracy, efficiency, and early detection of dental conditions in children. This review explores the current landscape of AI-driven technologies employed in the identification of pediatric dental diseases, including dental caries, malocclusion, developmental anomalies, and periodontal conditions. Various AI techniques, such as machine learning, deep learning, and convolutional neural networks (CNNs), are examined for their diagnostic potential and performance relative to traditional methods. The review also examines the integration of AI with radiographic imaging, intraoral scanners, and other diagnostic tools commonly used in pediatric dental practice. While AI presents considerable advantages such as speed, objectivity, and the potential to reduce human error, limitations, including data privacy, lack of standardized datasets, and ethical considerations, are also highlighted. Overall, this review underscores the ground-breaking potential of AI in pediatric dentistry and emphasizes the need for further research, validation, and clinical integration to realize its benefits fully.

## Introduction

Artificial intelligence, also referred to as the “fourth industrial revolution,” leverages computer systems to mimic human-like rational analysis, judgement and intelligent behaviour (1). AI methods have shown remarkable aptitudes and capacities for detecting meaningful data patterns, which has prompted extensive experimentation with these methods as tools in clinical trials, notably to support every stage of diagnosis, treatment and prognosis (2).

Within the realm of AI, Machine learning (ML) and Deep learning (DL) play pivotal roles in advancing medical and dental practices. ML enables systems to construct statistical models that aid in data comprehension and intelligent analysis (3, 4). Large datasets are used to train algorithms to recognize meaningful patterns, which serve as the basis for making decisions or forecasting results on new inputs (5). On the other hand, inspired by the way human brain operates, deep learning leverages artificial neural networks to process and learn from data. It relies on vast amounts of data and complex algorithms, often resulting in higher accuracy (3). Deep learning is further classified as an Artificial neural network and a Convolutional neural network.

A key goal in managing oral conditions in children is enabling practitioners to quickly detect the disease, evaluate its severity and choose the most appropriate treatment tailored to each patient. Conventional imaging methods like x-rays and CT scans play an essential role in diagnosis by offering detailed views of localised areas and comprehensive overviews of oral structures, respectively (6). In addition, cutting edge imaging tools like intraoral cameras and Optical Coherence Tomography(OCT) enable real time, non invasive visualization of oral tissues aiding in early detection of abnormalities (7–9). Artificial intelligence's capability to process and analyse large volumes of complex data—including patient records, radiographic images, clinical photographs, and other related data—makes it a valuable asset for generating diagnostic insights and planning treatments (10). AI models can combine information from radiography, intraoral imaging and patient records to detect and diagnose early childhood caries, malocclusion, trauma, gingival health and various other conditions, enabling earlier interventions (11).

AI technologies are increasingly being utilized in pediatric dental diagnostics, enabling early identification of oral pathologies, facilitating growth and development evaluations for orthodontic planning, quantifying plaque levels and distinguishing between supernumerary, primary and permanent dentition (6). Convolutional Neural Networks (CNNs), a type of Deep learning AI tool, are widely favoured for image classification because they can automatically learn and extract relevant features through successive layers of convolution and pooling. Their distinctive layered structure, which includes trainable filters, allows CNNs to perform exceptionally well in medical computer vision applications. As a result, they are often the top choice for AI-powered computer vision tasks in dental practices (12, 13).

The use of AI in pediatric dentistry signals the beginning of a new era of accuracy and productivity, with chances to improve patient satisfaction and diagnostic precision. Dental professionals will be able to use data-driven insights, automated procedures, and virtual consultation platforms to provide more individualized, proactive, and efficient dental care in the future as AI evolves. However, the risk of automation bias, the human tendency to over-rely on automated systems, even when those systems provide incorrect or misleading information, or when human judgment would be more accurate, is also looming large. This could lead to errors and potentially harmful outcomes.

This narrative review has attempted to analyse the various AI tools playing a role in the diagnosis of pediatric dental conditions, their accuracy compared to clinical assessments, and the future of AI in the precise diagnosis of pediatric oral diseases.

## Materials and methods

### Article eligibility criteria

This review included studies that focused on the use of Artificial intelligence in diagnosing dental diseases in pediatric patients. Artificial intelligence was defined broadly to encompass tools involving both machine learning and deep learning techniques. The pediatric population considered ranged from birth through adolescence, in accordance with the guidelines set by American Academy of Pediatric Dentistry (AAPD). PICO framework used: Population: Children/pediatric patients with suspected dental diseases; Intervention: Artificial intelligence based diagnostic tools; Comparator: Conventional diagnostic methods; Outcome: Diagnostic accuracy.

Studies that explored AI applications in treatment planning or predictive modeling were excluded. Only peer reviewed journal articles published in English were considered. Publications limited to abstracts without accessible full texts, literature reviews, conference abstracts and letters to the editor were not included in the review. Inclusion and Exclusion criteria (Table 1); and Logic grid (Table 2) are given in the tables below.

**Table 1:** Inclusion and exclusion criteria.

Inclusion criteria	Exclusion criteria
Population: Pediatric population 0–18 years of age	Articles with only abstracts and not full text
Intervention: Artificial intelligence	Other literature reviews
Comparator: Other traditional methods	Conference proceeding, letters to editor
Outcome: Sensitivity, specificity and accuracy of diagnostic outcomes of artificial intelligence	Articles with studies done involving adult population
Peer reviewed and published in English Language	AI tools in the application of treatment procedures, prediction models

**Table 2:** Logic grid.

Population	Index test	Reference test	Diagnosis of interest
Child Children Adolescent Pediatric Stomatognathic diseases Dental diseases Mouth and tooth diseases	Artificial intelligence AI Machine learning Machine intelligence Deep learning Neural network Computer Reasoning Computational intelligence Computer vision system	All other conventional diagnostic methods in pediatric dentistry	Sensitivity and specificity Diagnostic Accuracy F1 scores Precision Mean intersection over union(MIOU) ROC Curve Area under the Curve Positive predictive value Negative predictive value

**Figure 1:**
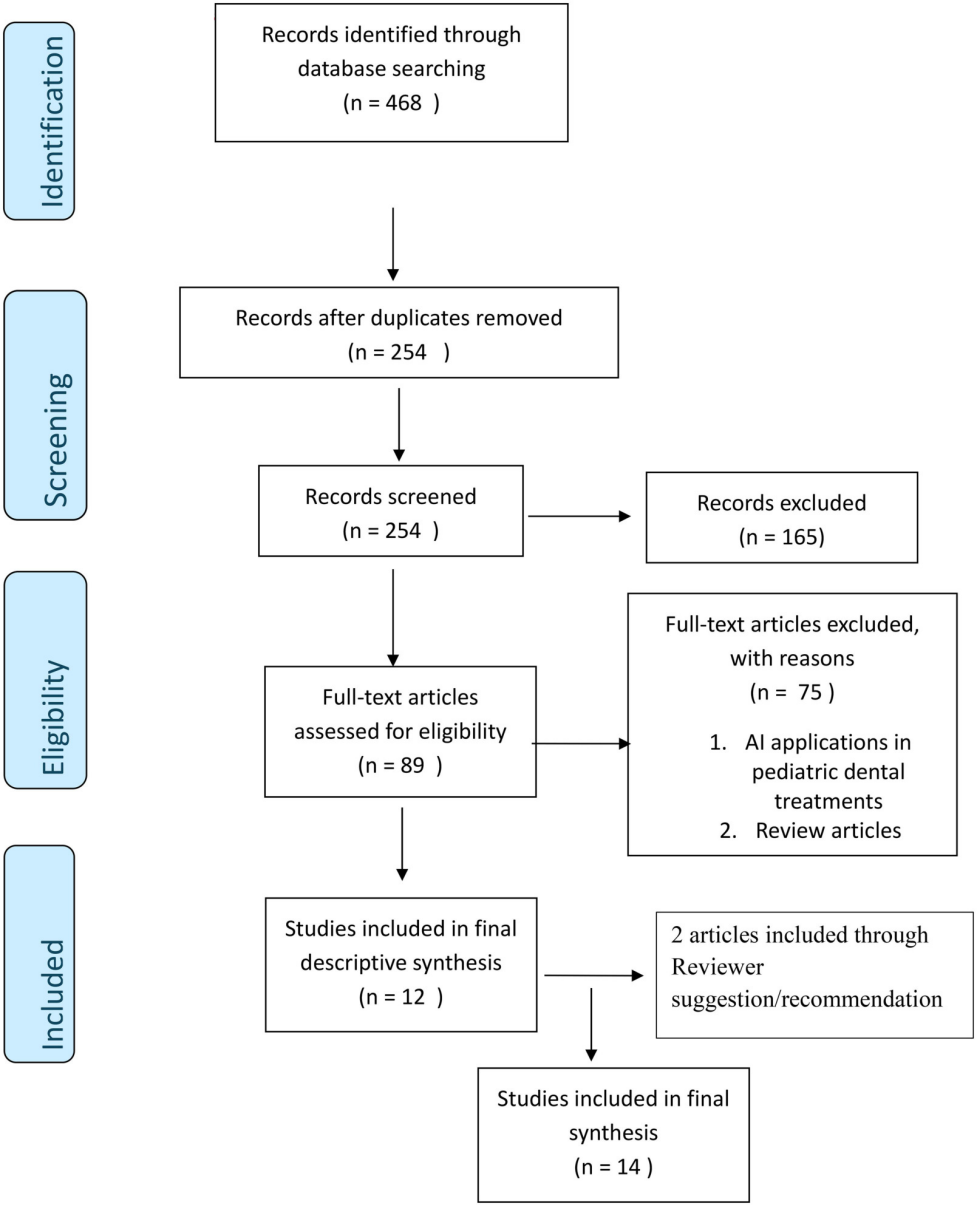
Flow-chart demonstrating methodology.

*Database sources and search strategies:* The search included the following databases: PubMed, Cochrane Library, EBSCO Dentistry and Oral Science Source, Google Scholar and Scopus. Keywords which were used in the search, refined using Boolean operators included, (“artificial intelligence” OR “machine learning” OR “deep learning”) AND (“oral diagnosis” OR “dental diagnosis”) AND (“child” OR “children”). Titles and abstracts were initially screened to assess relevance, followed by a comprehensive full-text review of the selected studies. The included articles were thematically categorized according to their primary focus areas—such as caries diagnostics, gingival disease detection, and applications in orthodontics, forensics and special needs dentistry—to ensure a structured and coherent presentation of the findings. Publication year range was from the year 2010–2025 and the date of last search was July 31st 2025 (Figure 1).

## Discussion

### Applications of artificial intelligence in the diagnosis of dental diseases

#### AI in dental caries detection

Dental caries, commonly known as tooth decay, remains one of the most prevalent oral health issues among children. Contemporary approaches to caries management prioritize prevention, non restorative interventions, and minimally invasive treatments. Detecting caries in its early stages is thus critical as it enables timely intervention and helps preserve healthy dental tissues (14–16).

In the 2019 study conducted by Al Kheraif et al. (17), the researchers successfully diagnosed and differentiated between tooth decay and acid erosion in five-year-old children using an advanced computational approach. They employed an evolutionary multi-objective cuckoo algorithm in conjunction with a Ruzzo–Tompaoptimised regulatory feedback neural network. This hybrid model demonstrated remarkable diagnostic accuracy, achieving 99.22% in identifying abnormal dental features and maintaining a low misclassification rate of just 1.2%. The high accuracy and low error rate underscore the potential of integrating bio-inspired algorithms with neural networks in pediatric dental diagnostics. Such systems can offer early, non-invasive, and highly reliable detection of dental issues, which is particularly beneficial in young children where clinical diagnosis may be more challenging. In a study by Xiao et al. (18), a smartphone application called AICaries was developed to assist in the early detection of Early Childhood Caries (ECC) in children aged 1–5 years. The app allows parents to use their smartphones to capture images of their children's teeth and identify early signs of caries, enabling timely intervention while the condition remains reversible.

Similarly, Li et al. (19) developed a diagnostic model based on the presence of 14 bacterial species, achieving a diagnostic accuracy of 83.08% and an AUC of 92.17%, highlighting its potential for universal caries detection.

These models still struggle to accurately identify cavities in molars and other teeth with intricate structures. Moreover, much of the existing research on automated caries detection is heavily centered on imaging, frequently neglecting important elements like patient history and clinical examination results that dentists normally consider while making a diagnosis (20, 21).

### AI in the diagnosis of gingival pathologies

Gingival disease is a condition triggered by the human immune system's inflammatory response to various bacterial species in the oral cavity. Early detection of this disease through artificial intelligence can significantly enhance oral health, ultimately leading to improved overall well-being and quality of life (22).

In a 2020 study by You et al. (23), researchers explored the use of artificial intelligence in pediatric dentistry by developing a conventional convolutional neural network (CNN) to detect dental plaque on primary teeth using intraoral photographs. The model was initially trained on a dataset of 886 high-quality intraoral images and then clinically validated on an additional 98 photos. To further evaluate its robustness, the model's performance was also compared to that of a dentist using 102 lower-resolution intraoral images.

Detection accuracy was assessed using the mean intersection-over-union (MIoU) metric, with the AI model achieving an overall MIoU of 0.726 ± 0.165. Notably, the AI model performed on par with or better than the dental professional across various scenarios—achieving MIoUs of 0.736 vs. 0.695 during the initial comparison, 0.689 after a one-week interval, and 0.724 vs. 0.652 on low-resolution images.

These results indicate that AI-powered plaque detection can match the diagnostic accuracy of a trained specialist, even when imaging conditions are suboptimal.

### AI in orthodontic diagnosis

Malocclusion often disrupts normal occlusal function, leading not only to physical discomfort but also to psychological distress, ultimately diminishing an individual's quality of life (24, 25).

Maxillary first molar Ectopic eruption is a common anomaly in pediatric dentistry often causing early loss of primary second molars, premolar impaction and arch length deficiency (26). While panoramic radiographs are valuable for detecting EE, accurate diagnosis heavily relies on the clinician's experience. In a study by Liu et al. (26) an AI based auto screening model was developed using panoramic radiographs selected from a cohort of 1,480 children aged 4–9 years. The model achieved a sensitivity of 86%, specificity of 90% and predictive values of 0.86 and 0.88—surpassing the diagnostic performance of three experience pediatric dentists in identifying ectopic eruption. With early and reliable detection of EE, clinicians can intervene sooner, potentially preserving the adjacent primary second molars, maintaining arch integrity and preventing the need for more invasive or complex orthodontic treatment later.

Dental age estimation is essential for assessing physical development and identifying abnormalities in children's growth. In a 2024 study by Shi et al. (27), a three-step automated system was developed to estimate dental age in children aged 3–15 years. This system first used a YOLOv3 network to detect and number teeth in panoramic radiographs, followed by a specially designed SOS-Net to determine tooth development stages using a modified version of the Demirjian method. The YOLOv3 model demonstrated a high mean average precision (mAP) of 97.50% for tooth detection, while SOS-Net achieved a staging accuracy of 82.97%. The complete framework estimated dental age with a mean absolute error of 0.72 years (excluding third molars), providing a fast, reliable, and precise method for identifying developmental issues in pediatric patients.

In a 2025 study by Ghorbani et al. (28), an advanced AI system was created to identify and number both primary and permanent teeth using occlusal photographs. The system employed two convolutional neural network (CNN) models: the first model detected the location and presence of teeth by generating bounding boxes, while the second model enhanced these detections by classifying the teeth and assigning tooth numbers. Developed in Python using YOLOv8, the system achieved a sensitivity of 99.89%, a precision of 95.72%, and an F1 score of 97.76%. Most inaccuracies occurred with less frequently represented tooth types, such as primary incisors and third molars. Among primary teeth, maxillary molars showed the best results, with precision over 94%, perfect sensitivity, and F1 scores above 97%. Conversely, the system performed poorest with mandibular primary canines, which had a precision of 88.52% and an F1 score of 93.91%. This research demonstrates major advancements in AI-driven diagnostic technologies for pediatric dental care.

### AI in pediatric forensics

One of the main goals in forensic science is to determine a person's age to help build a detailed biological profile. This is especially important in criminal investigations and mass disaster scenarios, where identifying individuals can be difficult due to incomplete or damaged skeletal remains (29). In forensic dentistry, estimating the age of sub adult individuals involves analyzing the growth and eruption of both baby (deciduous) and adult (permanent) teeth, along with examining the degree of root formation and the closure of the apical foramen (30). Bunyarit S.S et al(2020) employed an artificial neural network (ANN) approach using Demirjian's scores to develop an updated dental age classification. Their findings showed that these revised scores were effective in accurately estimating the ages of Malaysian and Chinese children and adolescents (31).

Artificial intelligence, particularly through the use of artificial neural networks, can be applied to dental imaging—such as x-rays—to estimate an individual's sex by analysing the dimensions, form, and developmental patterns of their teeth and jaw structures (32).

Tooth identification plays a vital role in forensic science due to the unique size, shape, and distinct groove patterns of each individual's teeth. The procedure entails matching dental records like x-rays, dental charts etc., with the teeth of the deceased to confirm their identity. Teeth are highly durable and often remain intact even under extreme conditions like fire or trauma, making dental identification one of the most reliable and efficient methods available (33).

### AI in the screening of supernumerary teeth

Panoramic radiographic screenings conducted by less experienced or younger dental practitioners may occasionally overlook the presence of supernumerary teeth (34). Despite these limitations, convolutional neural network (CNN)-based deep learning models have shown considerable promise in assisting with their detection. Ahn et al. (35) demonstrated the application of a deep learning approach for identifying mesiodens in primary and mixed dentitions, suggesting that such models can enhance diagnostic accuracy and efficiency, particularly for less experienced clinicians. Similarly, Kim et al. (36) developed a fully automated deep learning system capable of detecting the presence of mesiodens; however, the system faced limitations in accurately determining the exact number and anatomical location of the supernumerary teeth. Additionally, Kuwada et al. (37) reported on two deep learning algorithms designed to detect impacted supernumerary teeth in the maxillary region using panoramic radiographs. Nonetheless, challenges in precise identification persist, particularly in cases involving incomplete eruption of permanent teeth.

The study by Zaborowicz et al. evaluated a deep-learning approach for estimating chronological age in children aged 4–15 using quantitative measurements from panoramic dental radiographs. Instead of relying on traditional, subjective developmental charts, the authors extracted 21 geometric indicators from teeth and alveolar bone and trained neural network models on 619 pantomograms. The models demonstrated high accuracy, with mean absolute errors ranging from 2.34 to 4.61 months and R² values of 0.92–0.96, with the boys' model performing best. The method offered an objective and automated alternative potentially useful in forensic age assessment and pediatric dentistry, though its applicability is limited by its age range (4–15 years), exclusion of individuals with dental anomalies, and the need for validation on larger, more diverse populations (38).

Another study presented a new digital method to estimate the chronological age of children and adolescents (ages 4–18) by analyzing tooth and bone geometry from panoramic dental radiographs. Using a set of 21 quantitative “tooth geometry indicators,” the authors applied a metamodel combining Proper Orthogonal Decomposition (POD) for data dimensionality reduction with Gaussian processes (GP) for regression—hence “POD-GP”. The model was trained and tested on 619 radiographs and, after optimizing input data through POD (reducing to 7 amplitude features), achieved stable and relatively precise age predictions with a mean absolute error (MAE) about ± 7.5 months. Importantly, the method also provided a standard deviation for each estimate, offering a measure of confidence for individual age predictions. Compared to traditional analog dental-age charts (which may err by up to ± 12 months) and previous neural-network approaches, this POD-GP method appeared more accurate and offered the advantage of rapid, automated age estimation—useful in clinical, forensic, or adoption/immigration scenarios where precise age determination is needed (39).

### AI in special needs dentistry

Special needs dentistry, which serves individuals with special health care needs (SHCN), remains a notably underrepresented field within oral health care. Suboptimal dental health in this population can significantly affect overall well-being. A 2024 study by Rokhshad et al. assessed the diagnostic capabilities of nine AI chatbots in identifying conditions associated with syndromes pertinent to special needs dentistry. The chatbots achieved an average diagnostic accuracy of 55  ±  4% across all questions, with no statistically significant differences in performance among them. Although the responses were generally consistent, the study concluded that none of the evaluated AI tools met the threshold for clinical acceptability in the context of special needs dentistry (40) Table 3.

**Table 3:** Studies and their sensitivity and/or specificity.

Author(Year)	Country	Population	AI model used	Sensitivity/Specificity/ Accuracy
Al Kheraif et al. (2019) (17)	Saudi Arabia	5-year-old children	Neural network	Accuracy = 99.22%
Xiao et al. (2021) (18)	USA	1–5-year-old children	Clustering method	Qualitative findings
Li et al. (2021) (19)	China	6–8-year-old children	Random Forest	Accuracy = 83.08%
You et al. (2020) (23)	China	5–8-year-old children	Convolutional neural network	Mean intersection- over-union (MIoU) = 0.726 ± 0.165
Liu et al. (2022) (26)	China	4–9-year-old children	Deep learning	Sensitivity = 86% Specificity = 90%
Shi et al. (2024) (27)	China	3–15-year-old children	Deep learning	Accuracy = 82.97%
Ghorbani et al. (2025) (28)	Iran	Pediatric and adult patients	Deep learning	Sensitivity = 99.89%
Bunyarit et al. (2020)(31)	Malaysia	5–18-year-olds	Artificial neural network	Accuracy/sensitivity/specificity/precision not mentioned
Ahn et al. (2021) (35)	Korea	Children with mixed dentition	Deep learning	Accuracy ranged From 82% to 88% considering all AI models experimented
Kim et al. (2022) (36)	South Korea	Children with initial phase of primary dentition or mixed dentition	Deep learning	Mean accuracy, precision, recall, F1 score and AUC were consistently high, each measuring ≈ 0.971
Kuwada et al. (2020) (37)	Japan	Children with fully erupted incisors	Deep learning	Precision = 1.0
Zaborowicz et al. (2022) (38)	Poland	4–15 year old children	Neural network	Accuracy = 73%
Zaborowicz et al. (2022) (39)	Poland	4–18 year old children	Metamodel	Accuracy = 95%
Rokhshad R et al(2024)(40)	USA	–	ChatBots	Accuracy = 55%

## Limitations and ethical implications

AI integration in pediatric dentistry raises a number of ethical and behavioral issues that should be carefully considered. Key ethical concerns include patient privacy, data security, and obtaining appropriate informed consent—particularly relevant in minors where decision-making involves parents or legal guardians. Inadequately validated or poorly supervised AI systems may pose risks, including diagnostic errors or inappropriate treatment recommendations, underscoring the need for rigorous oversight (41).

From a behavioral perspective, AI technologies may have limitations in recognizing and responding to the emotional and developmental needs of young patients. Pediatric dental care often depends on empathy, nonverbal communication, and trust-building, which AI systems may not be able to replicate. Over-reliance on AI could potentially disrupt traditional patient–provider relationships, reduce human interaction, and affect a child's ability to develop coping and communication skills during dental treatment. Furthermore, AI might have trouble correctly interpreting nonverbal clues that are crucial for customizing pediatric care, such as anxiety, discomfort, or distress. While AI offers valuable support in diagnostics and clinical decision-making, it should serve as an adjunct rather than a replacement for human interaction in pediatric dentistry. Maintaining the presence and involvement of dental professionals and caregivers remains essential to ensure emotional reassurance, ethical integrity, and high-quality, patient-centered care (42, 43).

One big challenge for AI in pediatric dentistry is the bias and differences in the data used for training. Many AI models are trained on datasets that do not reflect the diversity of children around the world. These datasets are often derived from particular geographical areas, ethnicities or healthcare systems with more developed infrastructure, resulting in some cases low representation of children living in rural and underserviced environments as well as racial/ethnic minority populations. Therefore, AI models can have good performance in narrow environments but poor generalization to real-world clinical situations with a more diverse patient population. In addition, differences in image quality and imaging protocols, as well as diagnostic thresholds, make the results of AI not generalizable to other institutions (44).

### Impact on clinical generalizability of AI

Uneven ethnic representation of training data can have the following consequences: Morphological differences in primary and mixed dentition will not be captured, misclassification of normal anatomical variations as well as increased rates of false positives and false negatives (45). Differences in tooth eruption patterns may lead to flagging of normal eruption delays as abnormal, provide inaccurate risk assessments and cause misjudgements of certain conditions (46).

Where validation is still lacking:

1. AI accuracy vs. pediatric specialists
2. Performance across diverse ethnicities and tooth morphologies
3. Reliability on poor quality radiographs or uncooperative child images

## Challenges in implementation

There are tremendous technical and cost thresholds to using AI in pediatric dentistry, which has not been widely adopted. Moreover, there exists a lack of digital infrastructure in many dental clinics (particularly those in low-resource or rural settings), including poor-quality imaging systems and an absence of high-speed internet access, as well as compatible software platforms to support AI tools (43). In addition, the overall implementation and integration of AI into practice management systems may be complicated from a technical perspective as well as require significant IT support and staff training (44). The capital cost of entry for AI logistics (such as purchasing medications, disposal costs and the human resources) can be a significant barrier to smaller or private clinics/centres. Adding to these spending cuts is the fact that storage and cybersecurity expenditures escalate every year, while system enhancements have become a significant new cost factor (45). For public health or government-funded dental treatment facilities, such problems are especially severe. In many cases, budget constraints have been the norm for years without change. In the absence of financial incentives or policy backing, many professionals will be hesitant to adopt AI-derived results, or they will not be able to afford them. It means people with unequal access to modern care then bear unequal levels of economic and social costs, both individually as patients and in their communities. Therefore, conquering these technical and financial obstacles is of the utmost importance to achieve AI in pediatric dental practice that is both fair and widely available (46).

For many in the dental community, it is hard to trust an output automatically generated by a machine without human intervention; this also applies when the problem-solving process of AI models is not transparent. This so-called “black box” of mystery has made it hard for clinicians to trust AI recommendations, which is especially concerning when treating children, because clinical judgment involves subtleties and patient cooperation can vary. Furthermore, some providers may believe AI could erode their expertise or change how they practice medicine, which introduces resistance to use (47).

AI integration in pediatric dentistry for successful implementation would necessitate organised training and standardisation down the line. At present, a digital literacy gap occurs as the majority of dentists receive minimal or no formal education on AI technologies during their time in university. Lacking insight into how AI systems operate, process information and produce results, dentists may find it difficult or even risky to use them.

Today, the majority of dental professionals receive little to no formal training in AI technologies during their academic education; this creates a gap in digital literacy. However, the challenge remains that without some level of transparency and interpretability of how AI systems work, process information, and produce outputs, clinicians may not have the necessary tools to use such systems correctly or safely. In addition, there are no established protocols for incorporating AI tools into the clinical workflow, resulting in their use varying between institutions. Not only does this variability affect care quality, but it also hinders AI validation and performance comparison efforts across settings. To use these tools competently and ethically, schools and practitioners must develop clear guidelines on how and when to use AI and structured training programs to familiarize students with these tools (48).

## Future directions and opportunities

### Personalised pediatric dental care using AI

The application of Artificial intelligence is undoubtedly a game-changer, as it allows the process to move towards personalised and patient-centred treatment in pediatric dental care. When combined with electronic health records, imaging data, genetic information, and behavioural profiles, AI can help clinicians develop more personalised diagnostic and therapeutic strategies to meet the needs of individual children. Different unique risk factors such as dietary habits, oral hygiene patterns, socioeconomic status, and even salivary biomarkers can make a child more or less susceptible to certain dental conditions like early childhood caries or malocclusion, and AI systems can predict probability estimates of these conditions from vast datasets. The ability to predict child dental diseases enables early interventions and targeted preventive approaches to prevent the burden of advanced dental diseases in children. AI-based tools can also tailor communication and behaviour management strategies to the developmental and emotional capabilities of each child, leading to better cooperation and clinical results. Innovations in pediatric dentistry are directed towards more personalised care, and the emergence of AI as an assisted technology will not only make it efficient in clinical aspects but also will improve the quality of care in terms of patient-oriented context, providing us a step ahead towards precision dental medicine (49).

### Integration with other technologies

The evolution of AI and its synergy with emerging technologies including IoT, VR and AR are set to impact the future of pediatric dental care for the better. IoT-enabled devices (smart toothbrushes, wearable oral health devices, real-time monitoring devices) can pair with AI to monitor and transmit data about the child's oral hygiene behaviours, dietary patterns, and oral health in real-time, longitudinally. AI can analyse this real-time data, delivering specific patient feedback, alerts at an early stage of deterioration, and enhanced preventative planning. Likewise, these AI-enabled AR and VR technologies can be utilised for improved behaviour management, immersive distraction techniques, virtual dental education, and fear reduction in pediatric patients. Similar technologies can also help with clinician training by simulating complex pediatric cases. AI interfacing with IoT and immersive technologies can enhance interactive, data-driven, and patient-convenient pediatric dentistry and contribute to better clinical outcomes and patient experiences (50, 51).

VR distraction lowers anxiety, heart rate and cortisol in children during dental treatments. AI models detect real-time biosignals (heart rate, facial expression, movement) and automatically modify VR content intensity (calmer scene, interactive game, guided breathing) to down-tune anxiety or up-engage attention. Personalization keeps the youngster involved without overstimulation, minimizes physiological stress and increases compliance during injections and restorative therapy (52).

AI-driven pre-visit AR/VR exposure therapy helps to lessen dental phobia before the appointment. Based on the child's past answers and parent reports, a home/clinic AR or VR module utilizes AI to customize a graded exposure program (virtual “tell-show-do”), such as brief familiarization sessions. This reduces anticipatory apprehension and allows the kid to rehearse the clinic environment; fewer cancellations, improved first-visit behaviour, and decreased need for pharmacologic sedation (53). AI + AR for “smart” tell-show-do at chairside (augmented coaching for children and caretakers): AR goggles or tablet overlays provide a live, simplified animation guide on the child's own mouth or the dental chair (e.g., virtual toothbrush demo, animated step-by-step of injection), while an AI assistant tailors wording/pace to the child's age and measured fear. This makes behavioral guidance explicit and visually accessible for young children—promotes comprehension, decreases resistance, shortens process time, and increases treatment acceptability (54).

### Regulatory perspectives and global trends

With the advancement of Artificial Intelligence in pediatric dentistry, regulatory frameworks and worldwide tendencies have begun to shape how it is to be responsibly incorporated. Already, global agencies such as the U.S. FDA and EMA are writing adaptive regulatory pathways to inform and regulate AI-based medical tools, maintaining the safety, transparency, and performance of such devices (55). The regulations focus on the explainability of algorithms, privacy of the data, and the continuous monitoring post-deployment. In India, though not specific to AI in dentistry, strategy documents from the Ministry of Electronics and Information Technology (MeitY) and NITI Aayog, as the policy “think tank” of the Government of India, have paved the way for examining the moral and legislative considerations of AI in optometry (56). The Central Drugs Standard Control Organisation (CDSCO) will compare AI-based diagnostic software to medical devices in the future. Moreover, India's growing investment to address digital health infrastructure—including the National Digital Health Mission (NDHM)—could facilitate AI integration into practice, including dentistry. Nevertheless, there are hurdles to overcome, especially in the areas of data standardisation, practitioner training, and equal access in both rural and urban environments. These opportunities and challenges will require dental councils, regulators, and AI developers to work collaboratively to create a systematic approach that provides opportunities for trust, while maintaining patient safety, especially for children, a vulnerable patient population (57–63).

## Conclusion

Artificial Intelligence has potential promise for pediatric dental diagnosis because it can increase the accuracy, efficiency, and patient-centred approach of the diagnosis process. While AI has applicable resources in identifying early caries lesions or analysing growth patterns, its application across the continuum of care provides valuable information for practitioners who likely need to make more rapid and consistently valid decisions. AI is also not unencumbered by issues such as data bias, a lack of diverse and validated training datasets, and issues concerning clinical applicability, ethical issues, and resistance to adoption. For a country like India that has tremendous disparities in access to dental care, AI also needs regulatory clarity, practitioner training, and infrastructural integrity to ensure equitable access to AI possibilities. In the future, the ultimate potential for AI is in combination with emerging technologies, such as IoT and AR/VR, and its ability to lend itself to personalised and preventative care; with this, there are great opportunities to promote AI-based oral health interventions in pediatric dentistry. Ultimately, it will require collaboration between dental professionals and researchers, policy makers, and technology developers. With responsible implementation and continuous evaluation, AI will be a great asset in improving oral health for children, provided it is leveraged responsibly and continually monitored.

**Figure 2:**
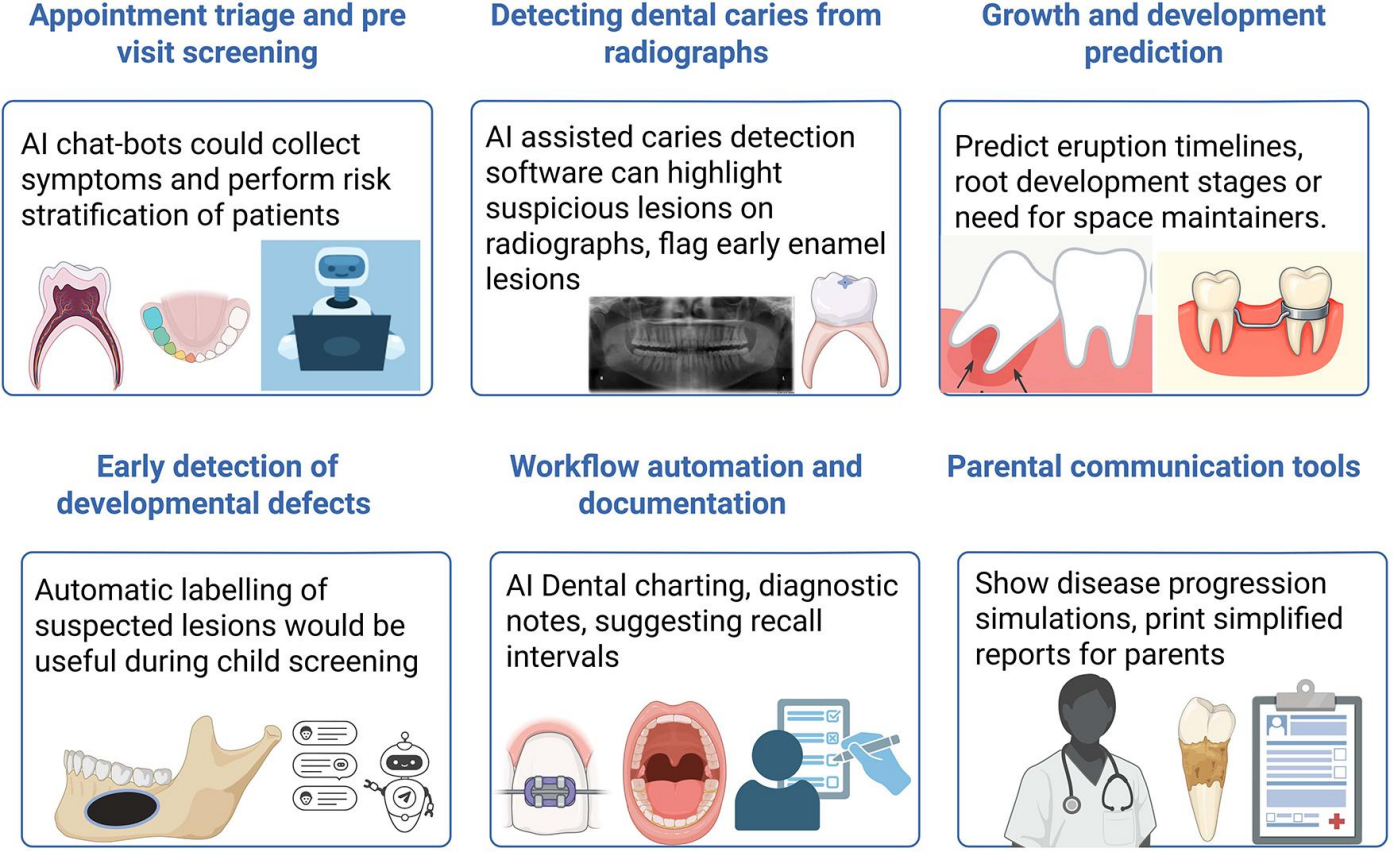
AI Tools integration into clinical workflow (59).

How AI Tools might be integrated into clinical workflow (Figure 2):

1. Appointment triage and pre-visit screening—AI chatbots coul00d collect symptoms and perform risk stratification of patients
2. Detecting dental caries from radiographs—AI assisted caries detection software can highlight suspicious lesions on radiographs before the dentist evaluates them, flag early enamel lesions that are easy to miss and could also generate risk score for the child based on radiographic patterns.
3. Growth and development prediction—AI Models could predict eruption timelines, root development stages or need for space maintainers. They could also analyse cephalometric radiographs for orthodontic planning.
4. Early detection of developmental defects—Automatic labelling of suspected lesions would be useful during child screening.
5. Workflow automation and documentation—AI Dental charting, generating standardized diagnostic notes, suggesting recall intervals based on diagnostic findings
6. Parental communication tools—Helps to show disease progression simulations, print simplified reports for parents.
